# Detection of gait impairment can enhance the early diagnosis of mild cognitive impairment and Alzheimer’s disease: A cross‐sectional study

**DOI:** 10.1002/alz70861_108448

**Published:** 2025-12-23

**Authors:** Boru Jin, Jinpan Zhang, Huayan Liu

**Affiliations:** ^1^ The First hospital of China medical university, Shenyang, Liaoning China; ^2^ The First Hospital of China Medical University, Shenyang, Liaoning China; ^3^ Department of Neurology, the First Hospital of China Medical University, Shenyang, liaoning China

## Abstract

**Background:**

This study aims to evaluate the diagnostic potential of gait parameters in differentiating healthy elders, mild cognitive impairment (MCI), and Alzheimer’s disease (AD).

**Method:**

263 individuals were enrolled in this study, involving 99 MCI patients, 61 possible mild AD patients, 46 possible moderate AD patients, and 57 controls (NC). Gait performance was detected through single‐task (normal speed and fast speed) and dual‐task (normal walking and counting backwards minus 3), recorded by three cameras (two on the side and one in the front). The RTMW model trained on the Cocktail14 dataset in the MMPose library was used to extract human key points from the captured gait videos. Kalman filtering was applied to remove high‐frequency noise, and the 'find peaks' function in the SciPy library was used to identify the lowest point of the footwork trajectory. Finally, we gained the gait parameters of stride speed (m/s), stride length (m), stride variation (%), and Dual‐task cost (DTC, %).

**Result:**

Firstly, in the single‐task, both normal and fast stride speed showed significant decline as the cognitive impairment aggravated, where there were statistical differences among the four groups (*p* <0.01). And the stride length of both normal and fast speed showed a significant decline in the possible AD (mild and moderate) group compared to controls (*p* <0.05). Secondly, in the dual‐task, stride speed significantly declined as cognition deteriorated, where the statistical differences can be observed between the MCI and control group (*p* <0.05), as well as possible AD (mild and moderate) and control group (*p* <0.01). Thirdly, fast stride speed in single‐task was the most optimal parameter in distinguishing MCI and control group (AUC=76.04%, specificity=60%, sensitivity=84.38%, Cut‐off=1.70m/s). Moreover, stride speed in single‐task exhibits great value in identifying AD from control, no matter normal stride speed (AUC=90.71%, specificity=80.3%, sensitivity=86.67%, Cut‐off=1.11m/s) or fast stride speed (AUC=90.35 %, specificity=77.27%, sensitivity=96.67%, Cut‐off=1.47m/s).

**Conclusion:**

Gait impairment can be significantly detected in MCI and possible AD, and stride speed and stride length would decline as cognition deteriorates. In single‐task, both normal and fast stride speed demonstrate optimal diagnostic efficacy in identifying both possible AD and MCI from controls.

